# Enhanced Metabolic Stress Augments Ischemic Preconditioning for Exercise Performance

**DOI:** 10.3389/fphys.2018.01621

**Published:** 2018-11-15

**Authors:** Joshua T. Slysz, Jamie F. Burr

**Affiliations:** Department of Human Health and Nutritional Sciences, University of Guelph, Guelph, ON, Canada

**Keywords:** exercise, hypoxia, occlusion, cycling, metabolites

## Abstract

**Purpose:** To identify the combined effect of increasing tissue level oxygen consumption and metabolite accumulation on the ergogenic efficacy of ischemic preconditioning (IPC) during both maximal aerobic and maximal anaerobic exercise.

**Methods:** Twelve healthy males (22 ± 2 years, 179 ± 2 cm, 80 ± 10 kg, 48 ± 4 ml.kg^−1.^min^−1^) underwent four experimental conditions: (i) no IPC control, (ii) traditional IPC, (iii) IPC with EMS, and (iv) IPC with treadmill walking. IPC involved bilateral leg occlusion at 220 mmHg for 5 min, repeated three times, separated by 5 min of reperfusion. Within 10 min following the IPC procedures, a 30 s Wingate test and subsequent (after 25 min rest) incremental maximal aerobic test were performed on a cycle ergometer.

**Results:** There was no statistical difference in anaerobic peak power between the no IPC control (1211 ± 290 W), traditional IPC (1209 ± 300 W), IPC + EMS (1206 ± 311 W), and IPC + Walk (1220 ± 288 W; *P* = 0.7); nor did VO_2_max change between no IPC control (48 ± 2 ml.kg^−1^.min^−1^), traditional IPC (48 ± 6 ml.kg^−1^.min^−1^), IPC + EMS (49 ± 4 ml.kg^−1^.min^−1^) and IPC + Walk (48 ± 6 ml.kg^−1^.min^−1^; *P* = 0.3). However, the maximal watts during the VO_2_max increased when IPC was combined with both EMS (304 ± 38 W) and walking (308 ± 40 W) compared to traditional IPC (296 ± 39 W) and no IPC control (293 ± 48 W; *P* = 0.02).

**Conclusion:** This study shows that in a group of participants for whom a traditional IPC stimulus was not effective, the magnification of the IPC stress through muscle contractions while under occlusion led to a subsequent exercise performance response. These findings support that amplification of the ischemic preconditioning stimulus augments the effect for exercise capacity.

## Introduction

It has been demonstrated that brief periods of circulatory occlusion and reperfusion, or ischemic preconditioning (IPC), can act to improve exercise performance (Jean-St-Michel et al., 2011; Bailey et al., 2012). Multiple studies have demonstrated that IPC performed in the minutes to hours preceding aerobic (De Groot et al., 2010) or anaerobic (Patterson et al., 2015; Cruz et al., 2016)exercise can improve performance but there appears to be great variability in response and, at present, the magnitude and consistency of the IPC effect across populations is not clear for either aerobic (Bailey et al., 2012; Hittinger et al., 2014; Sabino-Carvalho et al., 2017) nor anaerobic (Lalonde and Curnier, 2014; Paixao et al., 2014) exercise. Contributing to the lack of clarity around IPC as an effective ergogenic aid is the fact that the physiological signaling stimuli and associated downstream responses remain incompletely characterized. Of the leading physiological theories, local hypoxia [leading to HIF-1 signaling (Eckle et al., 2008)] and metabolite accumulation [such as adenosine, bradykinin, ROS, and opioids (Cohen et al., 2000; Marongiu and Crisafulli, 2014)] have received considerable attention; however, the existence of a dose-response relationship or identification of a threshold to trigger the biochemical pathways leading to the IPC effect remain unconfirmed (Cohen et al., 2000; Marongiu and Crisafulli, 2014). Given the many variations of IPC methodology reported in the current literature (i.e., differences in duration and number of cycles, occlusion pressure, volume of restricted muscle mass, local exercising, or remote muscle group) defining a pattern of the most efficacious method remains a challenge.

The metaboreflex is a key factor in controlling sympathetic outflow during exercise (Alam and Smirk, 1937) and studies utilizing ischemia to amplify metabolites and provide increased afferent feedback have shown an elevated sympathetic outflow and blood pressure response (Rowell et al., 1991; Tschakovsky and Hughson, 1999). Provided that the accumulation of metabolites is adequate, IPC could promote metaboreflex-induced increases in sympathetic outflow and blood pressure, preparing the body for subsequent exercise. IPC alone, however, has not been shown to elicit a sympathetic response, whereas the combination of cyclic bouts of blood flow restriction-reperfusion and treadmill exercise at 65% heart rate max has (Sprick and Rickards, 2017). It remains unclear if this combination can lead to improvements in performance, but it is possible that a sufficient metabolic stimulus (intramuscular perturbation) of IPC may be a crucial factor to elicit the desired effect.

By combining IPC with light exercise, such as walking, the muscle contractions thus evoked could function to amplify the hypoxic and/or metabolic preconditioning stimulus. As exercising while under blood flow occlusion may not be feasible or practical in certain situations (e.g., limited mobility during travel or when other temporal or spatial limitations exist in warm-up), the passive technique of electrical muscle stimulation (EMS) may be a more suitable option to similarly combine muscle contractions with IPC. Thus, we were interested in attempting to amplify the IPC performance effect by combining IPC with either active walking or passive EMS to enhance the stimulus evoked during a single treatment session. Both the active and passive models represent possible pre-competition strategies to increase tissue level hypoxia and metabolite accumulation compared with IPC alone.

Ischemic preconditioning is most commonly performed using supra-arterial occlusion pressures, dictating that both arterial inflow and venous outflow are subsequently restricted. As such there is a direct, and perhaps unavoidable, link between a greater emphasis on anaerobic metabolism and metabolite accumulation under these conditions which is challenging to meaningfully disentangle (Scott et al., 2014). Therefore, the purpose of this study was to identify the combined effect of increasing tissue level oxygen consumption and subsequent metabolite accumulation on the ergogenic efficacy of IPC during both maximal aerobic and maximal anaerobic exercise. It was hypothesized that IPC combined with muscle contractions induced by slow walking or electrical muscle stimulation would augment the ergogenic IPC effect, as demonstrated by greater aerobic and anaerobic power outputs.

## Materials and Methods

### Subjects

Twelve healthy males (22 ± 2 years, 179 ± 2 cm, 80 ± 10 kg, 47.7 ± 4 ml.kg^−1^.min^−1^) volunteered to participate in this study which employed a randomized cross-over design. All participants were recreationally active non-smokers. Participants had no medical history of chronic disease and were safe to exercise as confirmed through completion of a PARQ^+^ screening questionnaire (Warburton et al., 2014). This study was carried out in accordance with the recommendations of the University of Guelph’s human ethics research board with written informed consent from all subjects. All subjects gave written informed consent in accordance with the Declaration of Helsinki. The protocol was approved by the University of Guelph’s human ethics research board (REB# 15SE019).

### Protocol and Measurements

**FIGURE 1:**
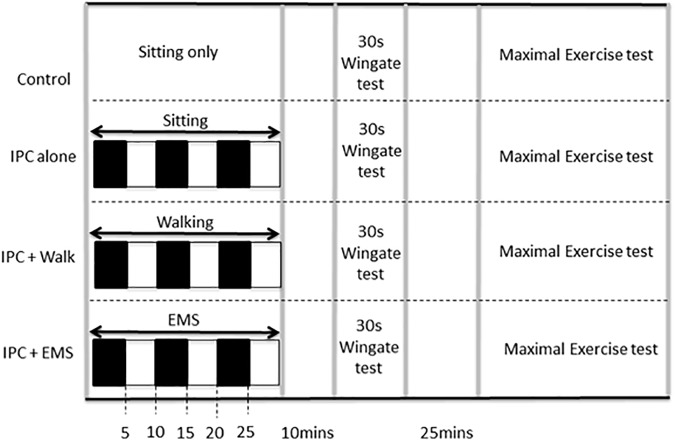
Study protocol including experimental and control visits, which were performed in random order. *IPC* ischemic preconditioning, *EMS* electrical muscle stimulation. Black boxes represent arterial occlusion on both the right and left leg, white boxes represent no occlusion.

All participants refrained from alcohol, caffeine, and intensive physical exercise for a least 24 h prior to testing. On each of four visits to the lab, participants performed both a 30 s anaerobic Wingate test with a standardized 5-min warm-up and warm-down and, after a 25-min rest, a subsequent incremental maximal exercise test. Both tests were completed on a cycle ergometer (Velotron Inc., Seattle, WA, United States). The four experimental visits were performed at least 1 week apart and at the same time of day. Each visit involved either (i) baseline control involving no IPC, (ii) traditional IPC, (iii) IPC in combination with EMS, and (iv) IPC in combination with treadmill walking (2 mph at 0% grade). Ten minutes following the IPC procedures (described below), the performance tests were initiated as per the graphical representation of the protocol presented in Figure 1. To eliminate possible training, learning, or familiarization effects, all conditions were assigned in a random order. Participants, who otherwise had little in the way of expectations concerning the expected effects, were blinded to all performance data and were not informed *a priori* as to the expected outcomes of the study to avoid introducing possible placebo or nocebo effects.

### Ischemic Preconditioning

Ischemic preconditioning was performed prior to exercise in a seated position using bilateral arterial occlusion. The occlusion cuffs (Zimmer ATS 1500; United States) were positioned around the proximal thigh and inflated to 220 mmHg for 5 min. This procedure, which is most commonly used in the IPC exercise performance literature (Incognito et al., 2016), promotes complete occlusion of both the arterial inflow and venous outflow in the lower limbs throughout the 5 min (Kooijman et al., 2008) as was confirmed in the present study using a near-infrared spectroscopy (MOXY, MN, United States), and the disappearance of a distal pulse. This ischemic procedure was repeated three times, each separated by 5 min of reperfusion (Incognito et al., 2016). IPC in combination with EMS was also performed in a seated position and involved the above-mentioned IPC protocol with electrically evoked muscle contractions throughout. The EMS (Compex International, Mi-Runner Sport, United Kingdom) involved two surface electrodes placed on both the Vastus Medialis and Vastus Lateralis at the distal and proximal position that best elicited a muscular contraction. Stimulation was applied using a pulse train length of 400 μs, delivered at a frequency of 50–100 Hz at a maximally tolerable intensity level. As participants accommodated to the stimulation during a session, the stimulation intensity was progressively increased. IPC in combination with walking involved the above-mentioned IPC protocol with slow walking on a standard motor driven treadmill (Sole F63 treadmill, Canada) at 2 mph (Sakamaki et al., 2011).

### 30 s Anaerobic Wingate Test

The 30 s Anaerobic Wingate test included a “flying start,” which consisted of 40 s of low load (100 W) pedaling prior to the introduction of the resistance (7.5% body weight), against which participants aimed to maintain maximal pedal revolutions for 30 s. Integrated Wingate testing software was used to calculate peak power output in watts.

### Incremental Maximal Aerobic Capacity Test

The incremental exercise test began with a resistance of 100 W and increased continuously at 1 watt every 3 s until exhaustion (i.e., the participant was unable to maintain a pedaling frequency of ≥50 rpm). Starting 1-min prior, and continuing throughout the maximal exercise test, oxygen consumption (VO_2_) was measured via indirect calorimetry using a face mask and optical turbine connected to a gas analyser with a sampling line (Cosmed Quark CPET, Rome, Italy). The maximal values were recorded as the highest reading that occurred after the data was smoothed using a rolling 30 s average. Attainment of true physiological max was confirmed for all subjects by the presentation of a plateau in VO_2_ (increase in ≤50 mL/min at VO_2_ peak and the closest neighboring data point), or respiratory exchange ratio (RER) ≥1.15 (Astorino et al., 2000). During the graded exercise test, VO_2_ at submaximal intensities were recorded and compared every 20 W between 120 and 200 W to investigate possible effects on submaximal exercise efficiency.

### Statistics

A Shapiro–Wilk test was used to confirm normality of data, prior to analysis. Comparisons between conditions were performed using repeated measures ANOVA, with LSD *post hoc* tests, as was appropriate. Statistical analyses were conducted using SPSS software (version 25; IBM, Chicago, IL, United States), with differences considered to be statistically significant at *P* < 0.05. All data is presented as mean ± SD, unless specified otherwise.

## Results

### 30 s Anaerobic Wingate Test

Peak anaerobic power was 1211 ± 290 W during the no IPC control and 1209 ± 300 W following traditional IPC. When IPC was combined with EMS and walking, peak anaerobic power was recorded to be 1206 ± 311 W and 1220 ± 288 W, respectively. There were no statistical differences between any groups (*P* = 0.7).

### Incremental Maximal Aerobic Capacity Test

**FIGURE 2:**
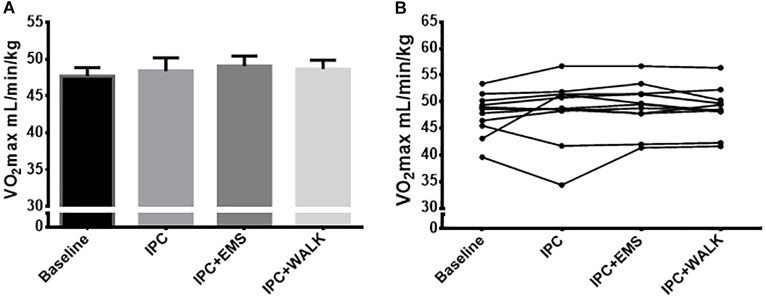
**(A)** Mean maximal oxygen uptake ml.kg^−1^.min^−1^ from the incremental maximal aerobic test for each intervention and baseline. **(B)** Maximal oxygen uptake ml.kg^−1^.min^−1^ from the incremental maximal aerobic test for each intervention and baseline. Data is presented as mean ± SE.

Baseline VO_2_max was 47.7 ± 4 ml.kg^−1^.min^−1^ and 48.4 ± 6 ml.kg^−1.^min^−1^ following traditional IPC. When IPC was combined with EMS and then walking, VO_2_max was recorded to be 49.1 ± 4 ml.kg^−1.^min^−1^ and 48 ± 6 ml.kg^−1.^min^−1^, respectively. There were no statistical differences between any groups (*P* = 0.3; Figures 2A,B). Submaximal oxygen consumption increased as the test progressed from 120 to 200 W, but these increases in VO_2_ every 20 W were similar in their pattern and magnitude across all conditions (Table 1).

**Table 1:** Oxygen consumption at submaximal exercise intensities during an incremental cycling test after no intervention (Control) ischemic preconditioning (IPC), ischemic preconditioning combined with electrical muscle stimulation (IPC + EMS), and ischemic preconditioning performed during slow walking at 2 mph (IPC + Walk).

	Control	IPC	IPC + EMS	IPC + Walk	*P*-value
VO_2_120 W (ml O_2_⋅kg^−1^⋅min^−1^)	25 ± 3	25 ± 4	25 ± 3	25 ± 2	0.9
VO_2_ 140 W (ml O_2_⋅kg^−1^⋅min^−1^)	28 ± 2	27 ± 4	28 ± 3	27 ± 3	0.5
VO_2_ 160 W (ml O_2_⋅kg^−1^⋅min^−1^)	30 ± 4	30 ± 4	30 ± 2	30 ± 3	0.8
VO_2_ 180 W (ml O_2_⋅kg^−1^⋅min^−1^)	33 ± 4	32 ± 4	33 ± 3	33 ± 3	0.5
VO_2_ 200 W (ml O_2_⋅kg^−1^⋅min^−1^)	35 ± 4	35 ± 4	36 ± 3	35 ± 3	0.8

*Data is presented as mean ± SD.*

**FIGURE 3:**
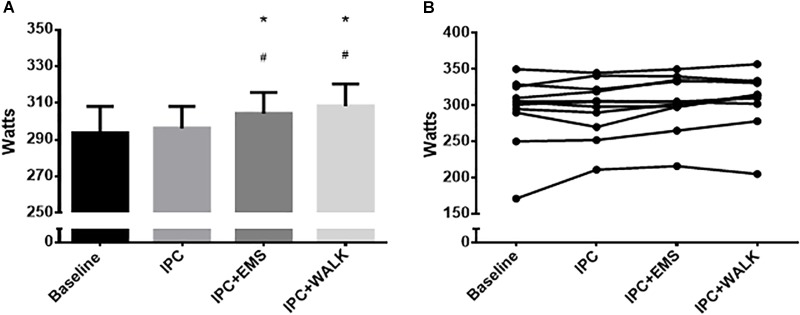
**(A)** Mean peak watts from the incremental maximal aerobic test for each intervention and baseline. Data is presented as mean ± SE and the differences were considered significant at *P* ≤ 0.05. ^∗^Represents statistically different from baseline; ^#^Represents statistically different from IPC alone. **(B)** Individual peak watts from the incremental maximal aerobic test for each intervention and baseline.

Peak watts, recorded at the point of exhaustion during the incremental maximal aerobic test, was 293 ± 48 W during the no IPC control and 296 ± 39 W following traditional IPC treatment. When IPC was combined with EMS and walking, peak watts increased to 304 ± 38 W and 308 ± 40 W, respectively (Figures 3A,B). Statistical analyses revealed significant increases in peak watts when combing IPC with EMS (*P* = 0.02) and walking (*P* = 0.03) compared to IPC alone. There were also significant increases in peak watts when combining IPC with EMS (0.04) and walking (*P* = 0.002) compared to the control group.

## Discussion

The present study sought to compare the effects of traditional IPC with an enhanced preconditioning stimulus, involving IPC combined with EMS or walking, for augmenting either aerobic and anaerobic performance. The main novel findings were that (1) IPC, when combined with walking or EMS significantly improved peak watt output in the maximal aerobic test to exhaustion, despite traditional IPC causing no significant benefit; (2) neither IPC nor an augmented adaptation of IPC improved maximal oxygen consumption; (3) neither IPC alone nor augmented IPC improved maximal anaerobic power. These findings suggest that a certain magnitude of metabolic and/or hypoxic stimulus may, thus, be important for stimulating the positive effects of IPC on exercise capacity, but that this effect was not driven by a change in aerobic or anaerobic maximal capacity.

### Exercise Performance

Previous studies have demonstrated increases in cycling peak power output (of 1.6–3.7%) following IPC treatment during maximal tests (De Groot et al., 2010; Crisafulli et al., 2011). The current data demonstrate traditional IPC to be ineffective for increasing peak power output during a maximal cycling test; however, when IPC was augmented with either passive twitches or active light-intensity muscular contractions, power output thereafter increased. More specifically, we observed a 3.8% increase in power with the addition of EMS to IPC and a 5% increase in power when slow walking was performed during the IPC treatment. The effect of an 11–15 W increase in max power could be quite meaningful in a competition situation, and when modeled using the current participants’ weight and the assumption of zero grade and wind while cycling, these augmentations in power would be expected to result in a 0.5–0.7 kph improvement in speed (Cycling Power Lab, 2018). While it is difficult to compare directly the muscular stress while under IPC, it is likely that the added stress of walking was greater than that of EMS. It is also likely that this utilized additional muscle mass, thus, the increased efficacy with walking is logical. Furthermore, it was observed that of the 12 participants, 8 did not initially demonstrate improvements in power output with traditional IPC. However, when a greater metabolic stress was imposed, 7 of the 8 “non-responders” became “responders” and increased maximal power output, which is in line with previous evidence that a greater physiologic stimulus reduces the rate of non-response to a given perturbation (Ross et al., 2015). Comparing to previous literature, it is worth noting that the one study which previously reported no change in peak power output during cycling following IPC also used the lowest occlusion pressure (Hittinger et al., 2014), and it is thus possible that the induced metabolic stress was lower, similar to the pattern we report here.

In line with the current model of increasing the accumulation of metabolic waste products, Crisafulli et al. (2011) have similarly attempted to magnify this effect by occluding leg circulation (for 3 min) immediately following submaximal cycling exercise. In partial agreement with our findings, this group reported that IPC consistently increased peak power output compared to a control test; however, augmenting the metabolic stress through a post-exercise occlusion did not demonstrate further benefit compared to traditional IPC. This may suggest that the initial IPC provided a sufficient stimulus to elicit an optimal performance effect, or that the addition of a brief 3-min augmented IPC period was insufficient to further amplify the response. In our study, in which we invoked muscle contractions throughout all cycles of the IPC, this stress was prolonged and repeated and may account for the differences in response. While we did not observe efficacy of traditional (using similarly matched) IPC protocol and graded cycling test with male participants, the addition of metabolic stress led to a response of similar magnitude. The discrepancy regarding the efficacy of traditional IPC between studies may be attributable to IPC protocol differences or participant training status, as subjects in the current study reached ∼10% higher peak watts, and thus a higher threshold of metabolic stress during IPC may have been required to elicit a similar response. The specific role of training status on the efficacy of IPC for affecting exercise performance requires further study.

### Maximal Aerobic Capacity

As is consistent with the majority of other studies, compared to the control group, there was no increase in VO_2_max after traditional IPC (Bailey et al., 2012; Hittinger et al., 2014; Sabino-Carvalho et al., 2017). Despite improvements in exercise performance (peak watts), when metabolic IPC stress was augmented with the addition of either passive or active muscle contractions VO_2_max remained unaltered compared to the control. This suggests that performance gains are not the result of an increase in maximal capacity. There was also no change in submaximal VO_2_ during cycling following traditional IPC, or following IPC combined with EMS or walking. This too is consistent with current literature (Clevidence et al., 2012) showing no change with traditional IPC, while also providing evidence that increasing the magnitude of the metabolic stimulus during IPC may have no effect on submaximal efficiency. Interestingly, 4 weeks of applying IPC after sprint interval training has been shown to increase VO_2_max (Taylor et al., 2016), suggesting an augmented IPC stress may be important in long-term aerobic adaptation rather than a short-term change. It must be recognized that it is possible our VO_2_ measures were affected by a preceding anaerobic test. If true, this is a potential explanation for the disagreement between a previous study (De Groot et al., 2010) that reported an increase in VO_2_max with traditional IPC; however, this is unlikely as our findings are consistent with the majority of the existing literature (Bailey et al., 2012; Hittinger et al., 2014; Sabino-Carvalho et al., 2017).

### Anaerobic Capacity

Using a standard 30 s anaerobic Wingate test, there was no change in anaerobic peak power following traditional IPC or following IPC combined with EMS or walking. This finding agrees with previous studies (Lalonde and Curnier, 2014; Paixao et al., 2014) showing no ergogenic effect of IPC on anaerobic exercise, while also providing novel evidence that the magnitude of the metabolic stimulus during IPC may have little impact on anaerobic exercise. A select few studies have shown a beneficial effect of IPC on anaerobic exercise (Patterson et al., 2015; Cruz et al., 2016), with positive effects typically occurring when IPC is employed further in advance (i.e., 30–60 min) of the exercise test; whereas studies that showed reduced or unchanged anaerobic performance used shorter periods (i.e., 5–15 min) (Lalonde and Curnier, 2014; Paixao et al., 2014) between IPC and the exercise test. Of note, the anaerobic test used in the current study occurred in the shorter time frame. The specific role of timing on the efficacy of IPC for affecting maximal anaerobic capacity needs to be further investigated. In addition, the studies that have shown positive effects of IPC on anaerobic exercise (Patterson et al., 2015; Cruz et al., 2016) appear to employ longer anaerobic effects (≥60 s) compared to the studies (Lalonde and Curnier, 2014; Paixao et al., 2014) that show no effect (30 s). The current study did not show any changes in peak or average power with IPC during the first or last 10 s of the 30 s Wingate test, suggesting that IPC does not assist with short-term energy provision. It is possible that IPC assists with energy provision with longer anaerobic efforts, but this remains speculative and requires further investigation.

The specific mechanism by which IPC works remains unclear. It is possible that the combination of IPC and rhythmic muscle contractions sufficiently altered local oxygen and metabolites to activate afferent feedback leading to a metaboreflex-induced sympathetic response during exercise, while IPC alone did not. This increase in sympathetic activity to non-active muscle could lead to greater blood flow and perfusion of the active muscle beds (Boulton et al., 2018), and if preconditioning were performed locally, the proper distribution of blood flow could be further aided by sympatholysis during treatment (Horiuchi et al., 2015). Nevertheless, we observed no change in whole body VO_2_max. An alternative explanation may be that IPC permits an enhanced central motor efferent command by attenuating inhibitory signals originating from metabolic sensory muscle afferents (Crisafulli et al., 2011). This would, thus, allow participants to exercise slightly beyond their individual critical threshold of exhaustion for the exercise, which fits with our finding of increased power. Indeed, a complete blockade of muscle afferent feedback during exercise, using an intrathecal administration of fentanyl, results in large increases in central motor drive and power output (Amann et al., 2011). de Oliveira Cruz et al. (2015) have observed an increase in aerobic energy provision with IPC, possibly reducing the utilization rate of anaerobic energy stores, lowering fatigue signals and delaying exhaustion. While the current study also does not offer any mechanistic insight, future studies will need to include more invasive measurements of blood flow, oxygen delivery, and arteriovenous oxygen difference across the working limb to determine whether IPC results in tissue specific improvements in these variables, which may be responsible for small improvements in peak watts.

### Limitations

As with most performance research, there were potential limitations to the current study that should be recognized. The inclusion of a sham control for each IPC intervention was omitted, both for practicality and to avoid introducing a potential training effect of excessive repeated testing of the same subjects. As such, it is possible that that a placebo effect could have occurred, if participants believed the treatment would help. However, participants were naïve to the expected treatment outcomes and it is conceivable that placebo effects were no more likely to occur than nocebo effects. It is undeniable that that this area of research, as a whole has struggled to find an effective sham control, and while previous research has used low-pressure sham conditions in which the cuff is only inflated to 10–20 mmHg (Jean-St-Michel et al., 2011; Bailey et al., 2012), this low pressure is easily distinguishable from true IPC. In addition, it is still unknown if the low-pressure itself can elicit a preconditioning response, thus we chose not to employ this technique in the current study and compared to a simple control condition. Finally, the current study was conducted with participants that are young and recreationally active, thus, the relevance of these interventions in an athletic or clinical population remain to be tested.

## Conclusion

In a group of participants for whom a traditional IPC stimulus was not effective, the amplification of an IPC stress through muscle contractions while under occlusion led to a subsequent increase in exercise performance. These findings support the hypothesis that there needs to be a sufficient metabolic and/or hypoxic stimulus for IPC to elicit an ergogenic action. From a practical standpoint, the addition of either passive or active muscle contractions to the standard IPC protocol of 3 sets of 5-min cycles of occlusion and reperfusion, may improve the efficacy and decrease “non-response” to IPC treatment, and this highlights that new variations of the IPC protocol should be explored in an effort to optimize the desired effect. Thus, augmenting the metabolic or hypoxic stress through muscle contractions may be an important and functional way to ensure the required metabolic/hypoxic stimulus is met for IPC to improve exercise capacity.

## Author Contributions

JB and JS had met the guidelines for authorship, and this manuscript had been read and approved by both authors.

## Conflict of Interest Statement

The authors declare that the research was conducted in the absence of any commercial or financial relationships that could be construed as a potential conflict of interest.
